# Construction of a ceRNA network of hub genes affecting immune infiltration in ovarian cancer identified by WGCNA

**DOI:** 10.1186/s12885-021-08711-w

**Published:** 2021-08-30

**Authors:** Rongjia Su, Chengjuan Jin, Lina Zhou, Yannan Cao, Menghua Kuang, Linxia Li, Jiangdong Xiang

**Affiliations:** Department of Obstetrics and Gynecology, Shanghai General Hospital, Shanghai Jiaotong University School of Medicine, 650 Xin Songjiang Road, Shanghai, 201620 China

**Keywords:** Immune infiltrates, Ovarian cancer, Hub gene, WGCNA

## Abstract

**Background:**

Ovarian cancer is the leading cause of death among gynecological malignancies. Immunotherapy has demonstrated potential effects in ovarian cancer. However, few studies on immune-related prognostic signatures in ovarian cancer have been reported. This study aimed to identify hub genes associated with immune infiltrates to provide insight into the immune regulatory mechanisms in ovarian cancer.

**Methods:**

Raw data and clinical information were downloaded from The Cancer Genome Atlas (TCGA) and University of California, Santa Cruz (UCSC) Xena websites. Single-sample gene set enrichment analysis (ssGSEA) and weighted gene co-expression network analysis (WGCNA) were used to identify hub genes. Kaplan-Meier analysis and differential expression analysis were applied to explore the real hub genes.

**Results:**

Through ssGSEA and WGCNA, 7 hub genes (LY9, CD5, CXCL9, IL2RG, SLAMF1, SLAMF6, and SLAMF7) were identified. Finally, LY9 and SLAMF1 were recognized as the real hub genes in immune infiltrates of ovarian cancer. LY9 and SLAMF1 are classified as SLAM family receptors involved in the activation of hematopoietic cells and the pathogenesis of multiple malignancies. Furthermore, 12 lncRNAs and 43 miRNAs significantly related to the 2 hub genes were applied to construct a lncRNA-miRNA-mRNA ceRNA network. The lncRNA-miRNA-mRNA ceRNA network shows upstream regulatory sites of the 2 hub genes.

**Conclusions:**

These findings improve our understanding of the regulatory mechanism of and reveal potential immune checkpoints for immunotherapy for ovarian cancer.

## Background

Ovarian cancer is the leading cause of death among gynecological neoplasms [1]. Cytoreductive surgery followed by platinum-based chemotherapy is the standard treatment for advanced ovarian cancer. Unfortunately, most patients experience recurrence and eventually die. Angiogenesis inhibitors such as bevacizumab and PARP inhibitors have been recently added to the available treatment schemes. In recent years, there has been increasing interest related to the role played by immunotherapy in ovarian cancer progression control.

Unlike chemotherapy or other targeted therapies, immunotherapies have the advantage of triggering the immune response, which clinically exerts specific, systemic, and durable antitumor effects. However, less than 15% of patients with advanced/metastatic ovarian cancer respond to immune checkpoint inhibitors [2, 3]. In other words, this toxic and costly treatment is potentially ineffective in the majority of ovarian cancer patients. Therefore, biomarkers affecting the immune response are needed to guide treatment decisions. Expression of programmed death ligand 1 (PD-L1) on the surface of T cells [4], the microsatellite instability (MSI) status [5] or DNA mismatch repair deficiency (dMMR) [6] and total tumor mutational burden (TMB) [7, 8] are the main predictive markers for the response to immunotherapy. However, because of the complexity of tumor-immune interactions, efforts to capture this complexity via a single analyte, such as PD-L1 expression or tumor mutational load, as a surrogate of potential tumor antigenicity, yield limited and incomplete information about the complex and dynamic nature of the tumor-immune microenvironment [9]. In summary, many efforts have been made to explore tumor immune signatures; however, few efforts to regulate tumor immune signatures have been reported.

With the development of high-throughput sequencing, bioinformatics has played an increasingly important role in the field of cancer research. Many studies consider only differences in the expression of genes between different samples and ignore the underlying connection of each gene. Weighted gene co-expression network analysis (WGCNA) [10] is a systematic biological method used to describe correlation patterns among genes in samples and can identify clusters (modules) of highly correlated genes for the investigation of clinical traits. In the present study, we performed WGCNA on RNA-seq data derived from The Cancer Genome Atlas (TCGA), reconstructed a gene (green) module related to immune infiltrates and identified hub genes in this green module.

To further reveal the regulatory mechanism of immune infiltrate-related hub genes, we constructed a lncRNA-miRNA-mRNA competing endogenous RNA (ceRNA) network. The ceRNA hypothesis states that a pool of long noncoding RNAs (lncRNAs), circular RNAs (circRNAs) and messenger RNAs (mRNAs) compete and bind to microRNAs (miRNAs), regulating their activity [11, 12]. Among the ceRNAs, miRNAs regulate the expression of their target genes by binding to the miRNA response elements on the target mRNAs, and lncRNAs act as molecular sponges to repress the negative regulation of target mRNAs by miRNAs. An R package called multiMiR was used to predict miRNAs for hub genes, and Star7Base [13] was used to predict interactions between lncRNAs and miRNAs. Finally, we identified 12 lncRNAs, 43 miRNAs and 2 mRNAs to construct a lncRNA-miRNA-mRNA ceRNA network.

Overall, in our study, we aimed to screen hub genes associated with immune infiltrates of ovarian cancer using the WGCNA method, construct a ceRNA network, and provide insight into the regulatory mechanisms of immune infiltrates in ovarian cancer.

## Methods

### TCGA data download

Level 3 HTSeq-Counts data, HTSeq-FPKM (per million fragments mapped) data and level 1 clinical information (shown in Table 1), such as age, histological type, survival and outcome of patients with ovarian cancer, were downloaded from The Cancer Genome Atlas (TCGA; http://cancergenome.nih.gov/). We chose “cystic, mucinous and serous neoplasms” in “Disease Type” as our research object. There were 379 samples from 376 ovarian cancer patients included in the TCGA program. Details on these samples are listed in Table 1. Because the TCGA database does not contain information on normal ovarian tissue, we also downloaded combined TPM (transcripts per kilobase million) data, including non-diseased ovarian tissue downloaded from the GTEx (Genotype-Tissue Expression) project, and ovarian cancer tissue from TCGA, and data from the UCSC Xena website (https://xena.ucsc.edu/), to compare the expression values of genes.

**Table 1:** Clinical information about TCGA-OV

	Alive	Dead	Overall
	(*N* = 147)	(*N* = 232)	(*N* = 379)
**Age (years)**			
Mean (SD)	57.1 (11.6)	61.1 (11.0)	59.5 (11.4)
Median [Min, Max]	57.0 [30.0, 87.0]	60.0 [36.0, 87.0]	59.0 [30.0, 87.0]
**Stage**			
I	0 (0%)	1 (0.4%)	1 (0.3%)
II	18 (12.2%)	5 (2.2%)	23 (6.1%)
III	111 (75.5%)	184 (79.3%)	295 (77.8%)
IV	16 (10.9%)	41 (17.7%)	57 (15.0%)
Missing	2 (1.4%)	1 (0.4%)	3 (0.8%)
**Sample**			
Primary Tumor	144 (98.0%)	230 (99.1%)	374 (98.7%)
Recurrent Tumor	3 (2.0%)	2 (0.9%)	5 (1.3%)

### Calculation of the Immunophenoscore

Data were analyzed in the R programming environment, version 4.0.2. The names of genes were converted from the Ensembl ID to the gene symbol through the AnnotationDbi and org. Hs.eg.db packages. The immunophenoscore (normalized enrichment score, NES) of each TCGA OV sample was calculated through the ssGSEA (single-sample gene set enrichment analysis) method using the GSVA package based on the expression of the representative genes in gene sets, which were downloaded from Cell Reports (10.1016/j.celrep.2016.12.019) [14]. ssGSEA ranks the genes by their expression in each sample and computes the enrichment score by integrating the differences between the empirical cumulative distribution functions of the gene ranks [15]. The results were visualized with the ggplot2 R package.

### Survival analysis

Univariate Cox regression analysis was performed to evaluate the association between the overall survival time and research objects, including the genes and immunophenoscores obtained above, using the survival and survminer R packages. *P* < 0.05 was considered to indicate a statistically significant difference. In addition, the log2(HR), 95% CI and statistical significance of the infiltrated immune cell types were calculated and illustrated using a forest plot through the forest plot package.

The patients were dichotomized based on the best cutoff of the immunophenoscore using the surv_cutpoint function of the survminer R package, which divides the immunophenoscore into high and low groups according to the best separation point and then generates a Kaplan-Meier curve. Log-rank tests were used to compare overall survival between different groups. The *P* values were adjusted for multiple testing based on the false discovery rate (FDR) according to the Benjamini-Hochberg method.

### Co-expression network construction

WGCNA is a systematic biological method used to build gene co-expression networks to mine network modules closely associated with clinical traits [10]. In the present study, we used the immunophenoscores of the infiltrated immune cell types in every sample as the target clinical traits. As read counts follow a negative binomial distribution, the RNAseq data of TCGA OV were normalized with the voom methodology of the limma R package [16]. This method estimates the mean variance of the log counts and generates a precision weight for each observation. The top 25% (6383) of genes with the highest median absolute deviation (MAD) used as a robust measure of variability were selected for WGCNA. Meanwhile, we removed 227 genes that were included in the gene sets we used.

Next, the average linkage method was performed for all pairwise genes to construct a co-expression similarity matrix. The co-expression similarity matrix was then transformed into the adjacency matrix by choosing the power of β = 4 as the soft-thresholding parameter to ensure an unsigned scale-free network. Then, we created a topological matrix using the topological overlap measure (TOM) [17]. To classify genes with similar expression patterns into gene modules, the dynamic hybrid cut method according to TOM-based dissimilarity was performed with the following major parameters: minModuleSize of 30 and deepSplit of 3. Finally, a cut-line (0.25) was selected for the module dendrogram, and some modules were merged according to the dissimilarity of estimated module eigengenes (MEs), which were defined as the first principal components of a given module and represent gene expression patterns in a module [18].

### Identification of clinically significant modules and hub genes

The interesting module was identified by calculating the relevance between clinical traits and MEs, which were the first principal components of a given module. Here, we chose the immunophenoscores of the infiltrated immune cell types as the clinical traits. The module that highly correlated with the target clinical trait was selected for further analysis.

Hub genes that were defined as highly interconnected with nodes in a module have been shown to be functionally significant. Three approaches were used to identify hub genes in this study. First, potential hub genes were defined by module connectivity (Pearson’s correlation of module membership > 0.8) and clinical characteristic relations (Pearson’s correlation of gene significance > 0.2). Module membership (MM), which quantifies how close a gene is to a given module, was referred to as the correlation between the ME and the gene expression profile. Gene significance (GS) was defined as the log10 transformation of the *p* value of each gene in the linear regression between gene expression and the clinical traits. Second, intramodule connectivity represents the relationship between genes within a specific module. The top 30 modules of interest with a certain intramodular connectivity value were identified as candidate hub genes. Genes with a connectivity degree ≥5 (the connectivity weight threshold was set to 0.25) in the co-expression network were defined as hub genes, and the modules of interest were constructed using Cytoscape 3.8.0 [19]. The genes with high MM, GS, intramodular connectivity and connectivity degree were considered hub genes in the module of interest.

### Pathway enrichment analysis

To further explore the biological significance of the hub genes, Kyoto Encyclopedia of Genes and Genomes (KEGG) pathway enrichment analysis was conducted on the hub genes based on the clusterProfiler package. An enriched pathway with a *p*-value ≤0.05 was considered to be statistically significant.

### Validation of hub genes

To further explore the prognostic value of hub genes in ovarian cancer, a survival analysis of hub genes was conducted using univariate Cox regression and Kaplan-Meier plotter, as mentioned above. To determine the expression values of hub genes in normal ovarian tissues and ovarian cancer tissues, we downloaded data from the UCSC Xena website, in which TPM values of TCGA and GTEx data were extracted, log2(x + 0.001) transformed, and combined. The hub genes with different expression levels and influence on survival were considered the “real” hub genes.

### CeRNA regulatory network

The lncRNA-miRNA-mRNA ceRNA network was constructed based on the ceRNA hypothesis that lncRNAs regulate the activity of mRNAs by sequestering and binding miRNAs, thereby acting as miRNA sponges. One R package called multiMiR was used to predict interactions between the mRNAs of hub genes and miRNAs. The multiMiR database [20] contains human and mouse data from 14 external databases that are categorized into three components: the three validated miRNA–target databases (miRecords [21], miRTarBase [22] and TarBase [23]), the eight predicted miRNA–target databases and the three disease−/drug-related miRNA databases. Only experimentally validated miRNAs were collected to construct the ceRNA network. StarBase [13] (http://starbase.sysu.edu.cn/) was used to predict interactions between lncRNAs and miRNAs. We chose lncRNAs whose ensemble IDs were included in the GENCODE database, those with a pancancerNum > 10 (pan-cancer types in which miRNA targets show anticorrelation relationships) and those with a clipExpNum > 0 (the number of CLIP-seq experiments) to construct the ceRNA network in Cytoscape 3.8.0 [19].

## Results

### Calculation of the Immunophenoscore and survival analysis

**Fig. 1:**
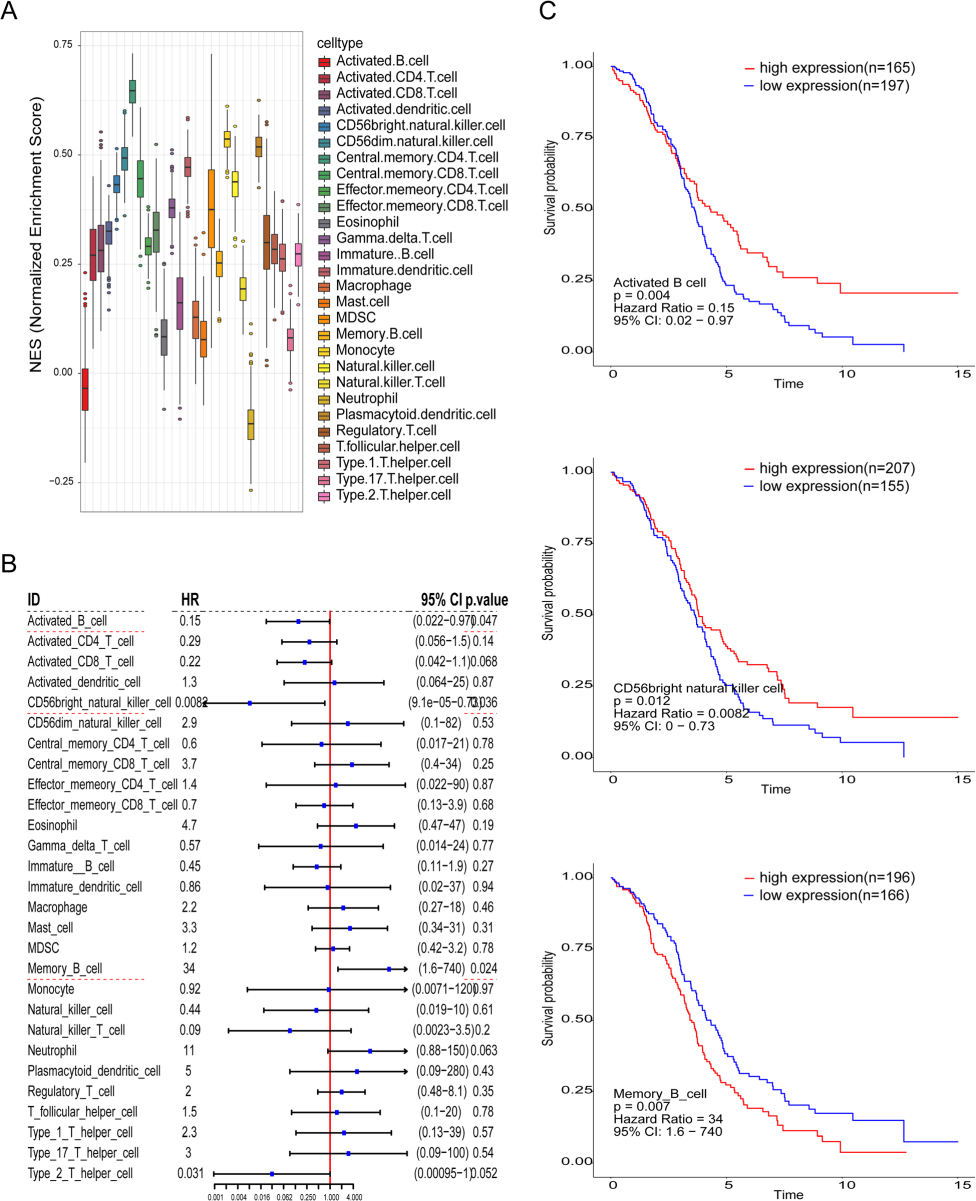
**A** Boxplot of 28 immune cell immunophenoscores in 379 TCGA-OV samples. **B** Forest plot of the hazard ratios of each infiltrated immune cell type for OS. Three significant infiltrated immune cell types are underlined with a red dashed line. **C** Kaplan-Meier curves for OS times. Survival analysis according to 3 infiltrated immune cell types. Red lines represent high immunophenoscores of the real hub genes, and blue lines represent low immunophenoscores

The immunophenoscores of 379 TCGA-OV samples were calculated through the GSVA package and used as clinical traits for the survival analysis and WGCNA. Because there were 374 primary tumor samples and 5 recurrent tumor samples, we extracted the immunophenoscores of the 374 patients with primary tumor for survival analysis and used all of them for WGCNA. The immunophenoscores of 28 infiltrated immune cell types from 374 primary ovarian cancer samples are shown in the boxplot in Fig. 1A.

To determine the predictive value of immune cell infiltration, we performed univariate Cox regression analysis. We found that three infiltrated immune cell types, namely, activated B cells (HR: 0.1500; *P* = 0.047), CD56 bright natural killer cells (HR: 0.0082; *P* = 0.036) and memory B cells (HR: 34.00; *P* = 0.024), were significantly correlated with OS (underlined with the red dotted line in the forest plot in Fig. 1B). Activated B cells and CD56 bright natural killer cells could be seen as protective factors; however, the HR (hazard ratio) of memory B cells was greater than 1, which means that this cell type is a poor prognostic factor. Figure 1C shows the Kaplan-Meier survival curves based on the best cutoff of the immunophenoscore in these three infiltrated immune cell types.

### Weighted co-expression network construction

**Fig. 2:**
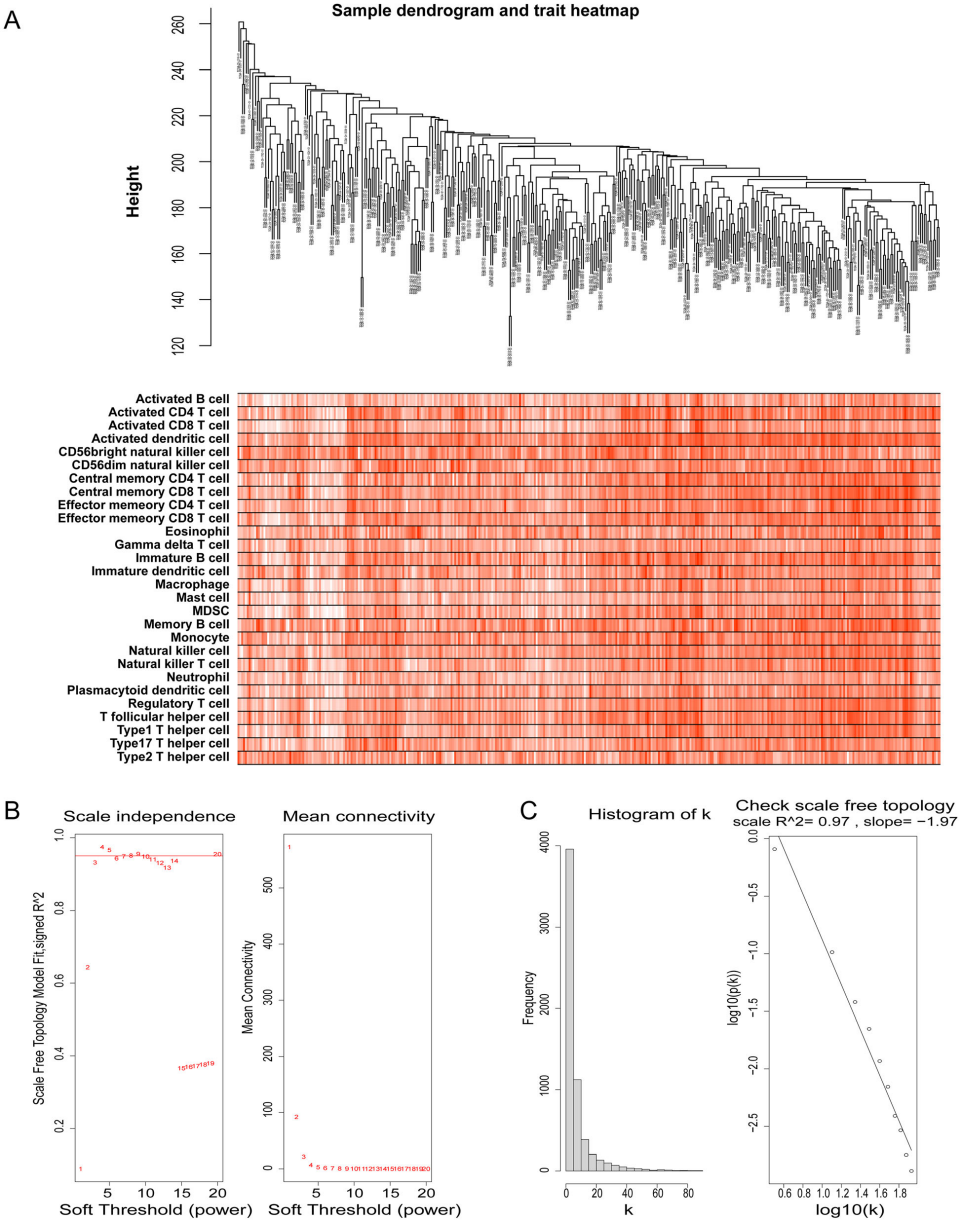
**A** Sample tree and trait heatmap. The clinical trait information includes the immunophenoscores of 28 infiltrated immune cell types in each sample. The larger the immunophenoscore is, the darker the color. **B** Analysis of the scale-free fit index for various soft-thresholding powers (β) and the mean connectivity for the soft-thresholding powers. **C** Histogram of connectivity distribution when β = 4 and determining the scale-free topology when β = 4

The 379 samples with clinical information (immunophenoscores of 28 infiltrated immune cell types) were clustered using the average linkage method and Pearson’s correlation method. The expression values of 6156 genes were used to construct the co-expression gene networks, and a sample dendrogram and trait heatmap were constructed (Fig. 2A). In this study, a power of β = 4 was selected to ensure a scale-free network (Fig. 2B, C) (scale-free R^2^ = 0.97, slope = − 1.97).

**Fig. 3:**
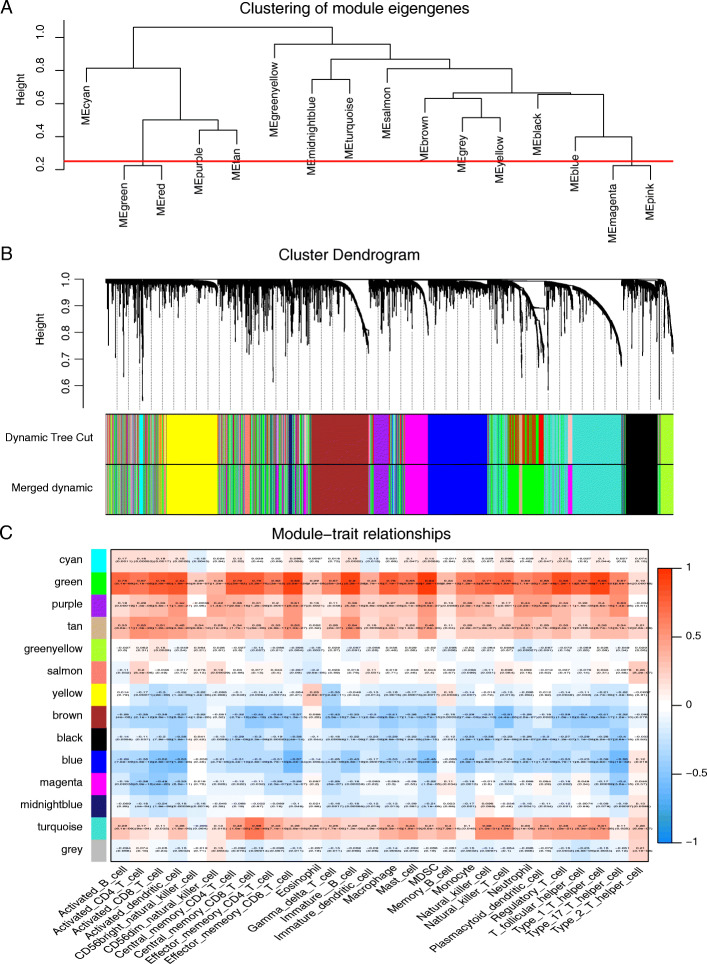
**A** Clustering of module eigengenes. A cut-line (0.25) was selected for the module dendrogram, and some modules were merged according to the dissimilarity of estimated module eigengenes. **B** A cluster dendrogram that presents 14 gene co-expression modules was built based on the dissimilarity of the topological overlap. The gray module indicates no co-expression between the genes. **C** Correlated heatmap of the adjacency of modules in the WGCNA. In the correlated heatmap plot, light blue represents low adjacency, while red represents high adjacency

After merging some modules through a cut-line (0.25) (Fig. 3A), a total of 14 modules were identified by the dynamic tree cut method. The clustering dendrograms of genes are shown in Fig. 3B. A heat map for the module-trait relationship is shown in Fig. 3C.

**Fig. 4:**
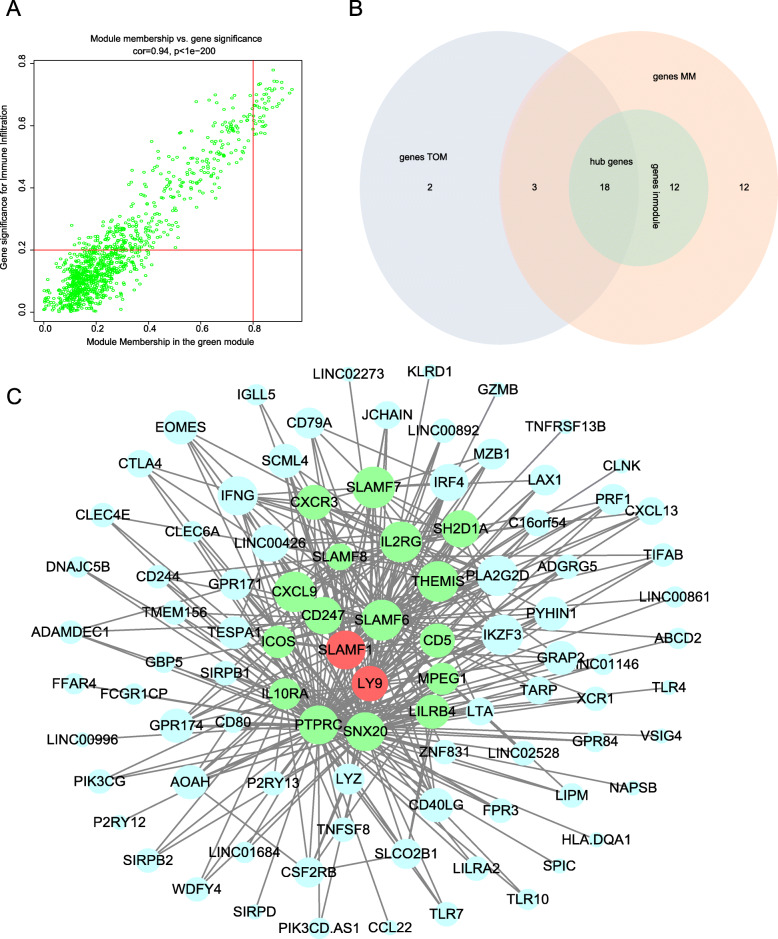
**A** Scatter plot of module eigengenes in the green module related to histologic grade. **B** Venn diagram. Selection of hub genes via WGCNA with 3 methods. **C** Visualization of the weighted gene correlation network in the green module. Red represents the real hub genes, green represents other hub genes identified through WGCNA, and light blue represents nodes in genes_TOM

Notably, the green module had the highest association with immune infiltration in the heat map of the module-trait relationship, and this module was selected as the clinically significant module for further analysis. However, only activated B cells among the three significant immune infiltration cell types had a strong correlation (0.75) with the green module. Therefore, 45 genes with high connectivity with activated B cells in the module, referred to as genes_MM, were selected based on the cutoff criteria (|MM| > 0.8 and |GS| > 0.2) (Fig. 4A). Meanwhile, we chose the top 30 genes with the highest intramodular connectivity, referred to as genes_inmodule. In addition, 23 genes were identified through Cytoscape (Fig. 4C) to have a connectivity degree ≥5 and a connectivity weight > 0.25 in the co-expression network; these genes were referred to as genes_TOM. Finally, 18 hub genes were identified as having high MM, GS, intramodular connectivity and connectivity degrees (Fig. 4B).

### Pathway enrichment analysis

**Fig. 5:**
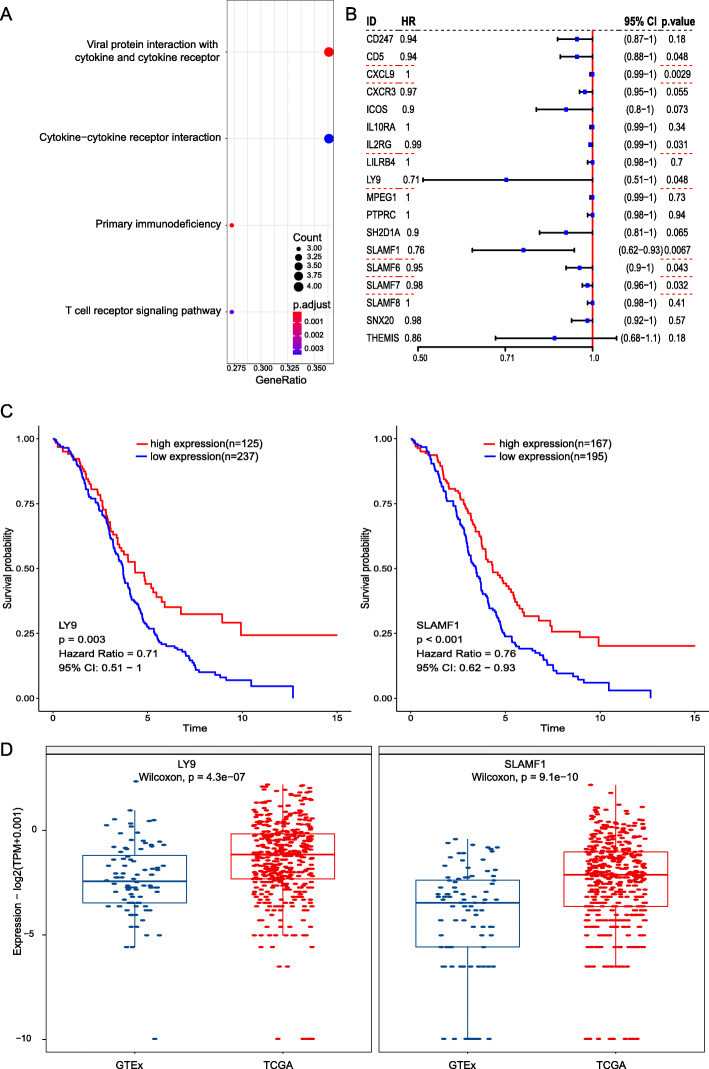
**A** Bubble diagram of the KEGG pathways analysis. **B** Forest plot of the hazard ratios of each hub gene for OS. Seven significant real hub genes are underlined with red dashed lines. **C** Kaplan-Meier curves for OS times. Survival analysis according to 2 real hub genes. Red lines represent high expression of the real hub genes, and blue lines represent low expression. **D** The expression of 2 real hub genes between TCGA and GTEx samples is shown in a boxplot. Red plots represent gene expression in TCGA samples, and blue plots represent gene expression in GTEx samples

According to Kyoto Encyclopedia of Genes and Genomes (KEGG) pathway analysis, our results demonstrated that these hub genes are mainly involved in the following pathways: cytokine−cytokine receptor interaction, viral protein interaction with cytokine and cytokine receptor, T cell receptor signaling pathway and primary immunodeficiency (Fig. 5A). These results indicate that the hub genes in the clinically significant module are mainly involved in the regulation of the immune system.

### Validation of hub genes

There were 7 genes among the 18 hub genes with statistical significance when conducting the survival analysis, as shown in the forest plot (Fig. 5B) (CD5, CXCL9, IL2RG, SLAMF6 and SLAMF7). All of them had *P* values < 0.05; however, their HRs were close to 1, which meant that these genes had little effect on overall survival (Fig. 5B). LY9 (HR = 0.71) and SLAMF1 (HR = 0.76) were selected as the real hub genes. Moreover, based on TCGA and GTEx data, the expression levels of these 2 real hub genes were significantly higher in tumor tissues than in normal tissues (Fig. 5C).

### A lncRNA-miRNA-mRNA ceRNA network is constructed

**Fig. 6:**
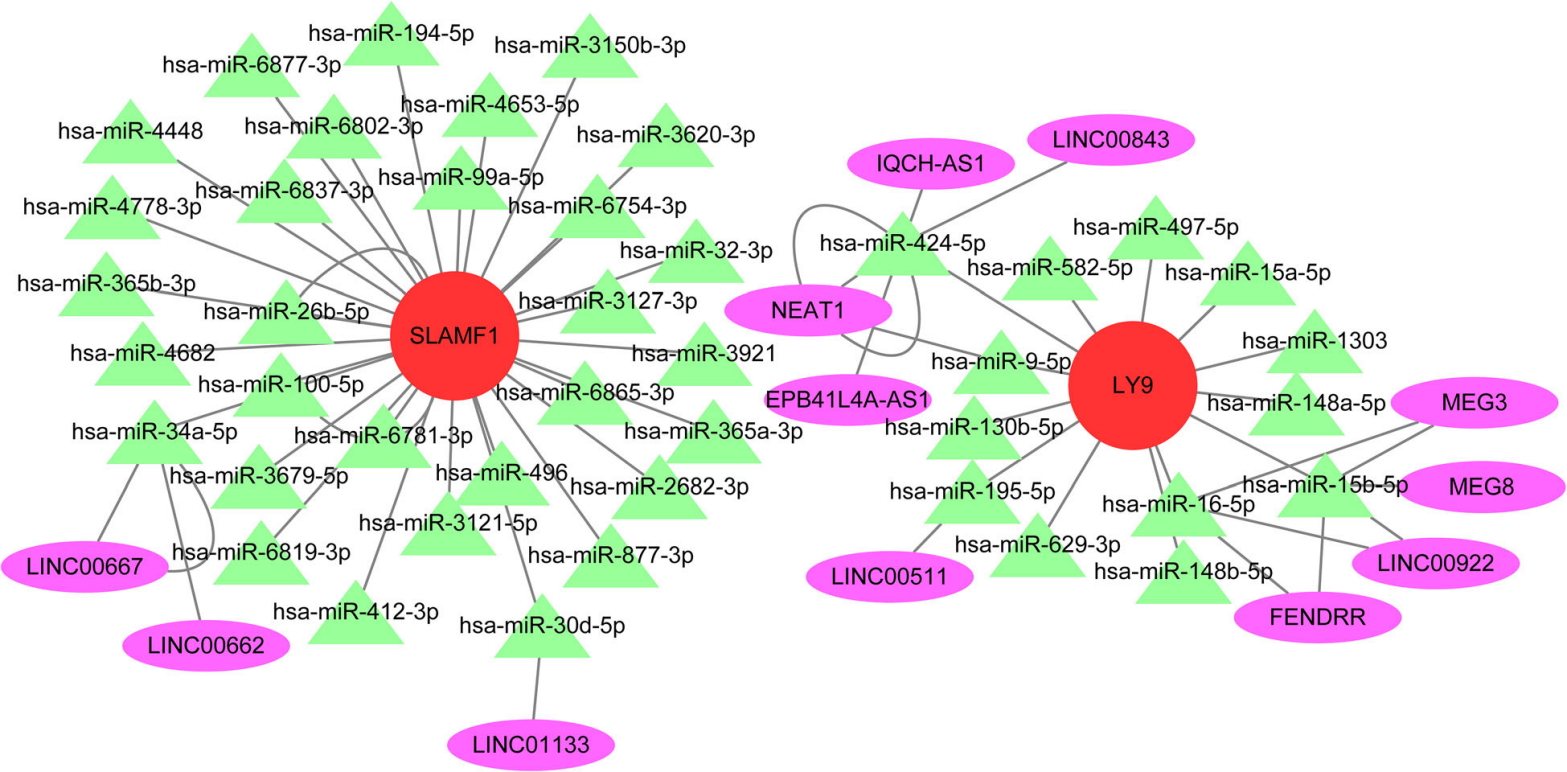
A lncRNA-miRNA-mRNA ceRNA network of immune infiltration in ovarian cancer was constructed with 12 lncRNAs, 43 miRNAs and 2 mRNAs

During the following step, we found that 43 experimentally validated miRNAs had relationships with the 2 hub genes. The network also included 12 lncRNAs that were obtained from StarBase [13] and connected with these miRNAs. Finally, a lncRNA-miRNA-mRNA ceRNA network was constructed in Cytoscape 3.8.0 and is shown in Fig. 6.

## Discussion

Ovarian cancer is one of the most lethal malignant gynecologic tumors worldwide [1] and is usually diagnosed at an advanced stage. Nearly all patients suffering from ovarian cancer inevitably undergo multistep development, such as occurrence, progression, regression, recurrence and chemotherapy resistance. Platinum-based chemotherapy remains the mainstay of treatment for ovarian cancer. In patients with recurrence, the platinum-free interval (PFI) is the most important factor for PFS and OS. The longer the platinum-free interval is, the higher the response rate (RR) and the longer the duration of response to treatment after first-line therapy [24]. There is an unmet need to prolong the PFI by maintenance therapy. PARP inhibitors are emerging as a promising maintenance treatment for high-grade serous ovarian cancers (HGSOCs) with germline or somatic BRCA1/2 mutations. The median progression-free survival time was approximately 36 months longer in the olaparib group than in the placebo group in the SOLO1 clinical trial [25]. Unfortunately, only approximately 20–30% of HGSOCs have BRCA1/2 mutations [26, 27]. The FDA approved bevacizumab, a humanized anti-vascular endothelial growth factor monoclonal antibody, in combination with chemotherapy for frontline and maintenance therapy for women with newly diagnosed ovarian cancer. However, the costly treatment and the use of bevacizumab during and up to 10 months after carboplatin and paclitaxel chemotherapy prolong the median progression-free survival by only 4 months in patients with advanced epithelial ovarian cancer [28]. Thus, new treatment strategies for ovarian cancer are urgently needed. Currently, immunotherapies such as anti-PD-1/PD-L1 antibodies are attracting attention worldwide. However, the response rate to anti-PD-1 antibodies is only 20–30% in various cancer types, and the response rate in ovarian cancer is lower than that in other malignancies (11.5–15%) [29]. Moreover, immune checkpoint inhibitors have serious adverse effects [30], such as diarrhea, rash, alopecia, pneumonitis, and hepatic or gastrointestinal adverse events. We urgently need new immune checkpoint inhibitors or to understand regulatory mechanisms targeted to ovarian cancer to improve the response rate and reduce adverse effects.

In this study, we calculated the immunophenoscores of 28 infiltrated immune cell types in cystic, mucinous and serous neoplasm ovarian cancer samples from the TCGA. Through univariate Cox regression analysis, we found that activated B cells, CD56 bright natural killer cells and memory B cells significantly influenced the survival prognosis of patients with ovarian cancer. Through WGCNA, we found that immune infiltration obviously had the highest association with the green module; however, only activated B cells among the three significantly infiltrating immune cell types had a strong correlation with the green module (0.75). Therefore, we selected activated B cells for further analysis. B and T lymphocyte attenuators (BTLAs) were reported to be identified mostly on B lymphocytes rather than on T lymphocytes and natural killer cells, and the combination of chemotherapy and the anti-BTLA antibody reduced the peritoneal tumor volume and extended survival in tumor-bearing mice [31]. Anne Montfort, Oliver Pearce and Eleni Maniati reported that B cells mainly infiltrated lymphoid structures in the stroma of high-grade serous ovarian cancer metastases and that there was a strong B-cell memory response directed at a restricted repertoire of antigens and the production of tumor-specific IgGs by plasma cells [32].

To further explore the genes related to the regulation of immune infiltration, we conducted WGCNA using the immunophenoscores of 28 infiltrated immune cell types as the target clinical traits. The green module was found to have the highest association with immune infiltration. Finally, 7 genes were screened from the module. However, the HRs of CD5, CXCL9, IL2RG, SLAMF6 and SLAMF7 were very close to 1. We selected LY9 and SLAMF1 as the real hub genes. LY9 (SLAMF3) and SLAMF1 are members of the signaling lymphocyte activation molecule family (SLAMF), which is a collection of nine surface receptors expressed mainly on hematopoietic cells. SLAMF receptors are expressed on B cells in the healthy and disease states and play a pivotal role in the control of malignant cell survival, interaction with cells in their tumor microenvironment, and retention in the supporting niches [33]. As shown in Fig. 5D, based on TCGA and GTEx data, the mRNA expression levels of LY9 and SLAMF1 were significantly higher in tumor tissues than in normal ovarian tissues. Moreover, as shown in Fig. 5C, the expression of LY9 and SLAMF1 was significantly associated with the overall survival of ovarian cancer patients. Thus, the expression of LY9 and SLAMF1 was higher in ovarian cancer tissues than in normal ovarian tissues, and the high expression of these 2 genes was relevant to the prognosis of ovarian cancer. Because they are mainly expressed on hematopoietic cells, they are a major research topic in the field of leukemia. The SLAMF1 receptor was reported to be an important modulator of the BCR (B cell receptor) signaling axis and may improve immune control in chronic lymphocytic leukemia by interfering with NK cells [34]. SLAMF1-deficient cells are resistant to drugs that activate autophagy, and these results indicate that SLAMF1 expression potentially affects drug responses in chronic lymphocytic leukemia [35]. LY9 overexpression in cancerous cells could represent a potential therapeutic strategy to improve the drug sensitivity of resistant cells [36]. These results indicate that LY9 and SLAMF1 might be potential therapeutic targets of ovarian cancer.

Accumulating evidence has indicated that ceRNAs are involved in the occurrence and development of cancer. Here, we identified 12 lncRNAs, 43 miRNAs and 2 mRNAs to construct a lncRNA-miRNA-mRNA ceRNA network. MiRNAs are the center of the ceRNA network, and all of these miRNAs were experimentally validated. Hsa-miR-15b-5p was reported to have a significantly higher expression level in ovarian cancer tissue than in normal tissue [37]. LINC00511 is highly expressed in breast cancer and correlated with a poor prognosis [38]. Junjie Zhang also predicted that LINC00511 bound to hsa-miR-195-5p in a ceRNA network of hepatocellular carcinoma [39]. LINC00511 plays an oncogenic function by interacting with EZH2 and repressing P21 expression in ovarian cancer cells [40]. The lncRNA NEAT1 regulates the proliferation and apoptosis of ovarian cancer cells, providing a potential therapeutic approach for ovarian cancer [41]. LINC00662 is highly expressed in ovarian cancer tissues, and the increased expression of LINC00662 is associated with short overall survival [42]. LINC01133 was reported to repress ovarian cancer cell proliferation, invasion, migration, and tumorigenic ability [43]. MEG3 regulates the PTEN gene in ovarian cancer cells to prohibit cell proliferation, promote apoptosis and block cell cycle progression [44]. Although our study lacked in vivo and in vitro validation, the results of our study provide possible prognostic markers for immunotherapy for ovarian cancer.

## Conclusions

Immune infiltrating activated B cells are associated with the survival prognosis of ovarian cancer patients. Moreover, LY9 and SLAMF1 were identified as the real hub genes associated with the overall survival of ovarian cancer patients by affecting the infiltration of activated B cells. LY9 and SLAMF1 might be potential therapeutic targets of ovarian cancer. The lncRNA-miRNA-mRNA ceRNA network demonstrates the molecular regulatory mechanism of the 2 hub genes. These findings improve our understanding of the regulatory mechanism of and provide potential therapeutic targets for immunotherapy for ovarian cancer.

## Data Availability

The datasets used and/or analyzed during the current study are available from the corresponding author on reasonable request.

## Abbreviations

TCGA: The Cancer Genome Atlas
UCSC: University of California, Santa Cruz
ssGSEA: Single-sample gene set enrichment analysis
WGCNA: weighted gene co-expression network analysis
PARP: Poly (ADP-ribose) polymerase
PD-L1: programmed death ligand 1
ceRNA: competing endogenous RNA
lncRNAs: long noncoding RNAs
circRNAs: circular RNAs
mRNAs: messenger RNAs
miRNAs: microRNAs
NES: normalized enrichment score
FDR: false discovery rate
MEs: module eigengenes
GS: Gene significance
KEGG: Kyoto Encyclopedia of Genes and Genomes
GTEx: Genotype-Tissue Expression
BTLAs: B and T lymphocyte attenuators
SLAMF: signaling lymphocyte activation molecule family
BCR: B cell receptor
MAD: median absolute deviation
OS: Overall Survival
PFS: Progression Free Survival
